# Evaluation of plitidepsin in patients with primary myelofibrosis and post polycythemia vera/essential thrombocythemia myelofibrosis: results of preclinical studies and a phase II clinical trial

**DOI:** 10.1038/bcj.2015.5

**Published:** 2015-03-13

**Authors:** A Pardanani, A Tefferi, P Guglielmelli, C Bogani, N Bartalucci, J Rodríguez, S Extremera, I Pérez, V Alfaro, A M Vannucchi

**Affiliations:** 1 Division of Hematology, Department of Medicine, Mayo Clinic, Rochester, MN, USA; 2 Department of Experimental and Clinical Medicine, University of Florence, Careggi, Firenze, Italy; 3 PharmaMar, Clinical R&D Department, Colmenar Viejo, Madrid, Spain

## Abstract

Previous data established that plitidepsin, a cyclic depsipeptide, exerted activity in a mouse model of myelofibrosis (MF). New preclinical experiments reported herein found that low nanomolar plitidepsin concentrations potently inhibited the proliferation of JAK2V617F-mutated cell lines and reduced colony formation by CD34+ cells of individuals with MF, at least in part through modulation of p27 levels. Cells of MF patients had significantly reduced p27 content, that were modestly increased upon plitidepsin exposure. On these premise, an exploratory phase II trial evaluated plitidepsin 5 mg/m^2^ 3-h intravenous infusion administered on days 1 and 15 every 4 weeks (q4wk). Response rate (RR) according to the International Working Group for Myelofibrosis Research and Treatment consensus criteria was 9.1% (95% CI, 0.2–41.3%) in 11 evaluable patients during the first trial stage. The single responder achieved a red cell transfusion independence and stable disease was reported in nine additional patients (81.8%). Eight patients underwent a short-lasting improvement of splenomegaly. In conclusion, plitidepsin 5 mg/m^2^ 3-h infusion q4wk was well tolerated but had a modest activity in patients with primary, post-polycythaemia vera or post-essential thrombocythaemia MF. Therefore, this trial was prematurely terminated and we concluded that further clinical trials with plitidepsin as single agent in MF are not warranted.

## Introduction

Primary myelofibrosis (PMF) and post-polycythaemia vera (post-PV MF) or post-essential thrombocythaemia myelofibrosis (post-ET MF) comprise a heterogenous group of chronic myeloproliferative neoplasms with no curative therapeutic modality at present except for allogeneic stem cell transplantation.^1, 2, 3^ They are characterised by expansion of a clonal haematopoietic stem cell population leading to a bone marrow fibrosis and impaired haematopoiesis resulting in severe anaemia, massive splenomegaly and extramedullary haematopoiesis along with the presence of severe constitutional symptoms. At present only one drug, ruxolitinib, has been approved primarily based on its ability to reduce splenomegaly and improvement of disease-related symptoms.^4, 5^ Therefore, agents with activity in this group of malignancies are needed.

Plitidepsin (Aplidin) is a cyclic depsipeptide originally isolated from the Mediterranean tunicate *Aplidium albicans* and currently produced by chemical synthesis.^6^ Plitidepsin was evaluated in a murine model of myelofibrosis (MF), the Gata-1(low) mice.^7^ Treatment with plitidepsin increased the platelet count in blood and marrow cellularity in the femur, and reduced the vessel density and expression of transforming growth factor-beta, vascular endothelial growth factor and thrombopoietin.^8, 9^ Therefore, plitidepsin ameliorated some of the traits of the myelofibrotic phenotype expressed by Gata-1(low) mice. In particular, the observed inhibition of transforming growth factor-beta and vascular endothelial growth factor expression, associated with reduced microvessel density, suggested a possible activity of plitidepsin in human MF, where levels of these two cytokines are abnormally increased.^8, 9^ The aforementioned data supported this drug as candidate for clinical evaluation in MF. Consequently, an exploratory phase II clinical trial was designed to evaluate the efficacy and safety of plitidepsin in patients with PMF, post-PV MF or post-ET MF (ClinicalTrials.gov identifier: NCT01149681). We also report herein new preclinical data obtained in cellular models of MF, including cell lines and primary patients' cells.

## Materials and methods

### Preclinical studies

Plitidepsin was provided by PharmaMar, dissolved in DMSO and stored in aliquots at −20 °C. For *in vitro* studies, we used the following human cell lines: HEL, UKE-1 and SET2 (JAK2V617F mutated) and K562 (*BCR/ABL1* mutated), and the murine Ba/F3 cell lines overexpressing the wild-type or V617F-mutated JAK2. Primary cells were obtained from patients with PMF, diagnosed according to the 2008 World Health Organization (WHO) criteria, under a protocol approved by the Institutional Review Board of Azienda Ospedaliera-Universitaria Careggi and after obtaining an informed consent. Normal CD34+ cells were obtained from healthy donors for transplantation purposes who agreed to donate the excess CD34+ cells, after providing an informed consent. Research was carried out according to the principles of the Declaration of Helsinki.

The drug-induced inhibition of cell growth by plitidepsin in human and mouse cell lines were measured by both a short-term microwell proliferation assay and a long-term clonogenic assay in agar. Quantification of apoptotic cells and assessment of the cell cycle distribution was accomplished by flow cytometry. Colony formation by CD34+ cells from MF patients and healthy controls in the presence of plitidepsin was measured in methylcellulose media for burst forming unit erythroid (BFU-E) and colony forming unit granulocyte-macrophage (CFU-GM) and in Megacult Collagen and medium with lipids for colony forming unit-megakaryocyte (CFU-Mk). The effects of plitidepsin exposure on the expression and phosphorylation of intracellular proteins were evaluated by western blot electrophoresis. Measurement of selected messenger RNAs (mRNAs) was performed by real-time PCR. A detailed description of the methods employed is provided in Supplementary Material.

### Patients

Patients were recruited at one investigational site each in the USA and Italy. The study protocol was approved by the Independent Local Ethics Committee of each participating centre and was conducted in accordance with the Declaration of Helsinki, Good Clinical Practice guidelines and local regulations on clinical trials. Signed informed consent was obtained from all patients prior to any study-specific procedure.

### Eligibility criteria

Eligibility criteria included patients ⩾18-years old with diagnosis of PMF or post-polycythemia vera (PV) MF or post-essential thrombocythemia (ET) MF, as per the revised World Health Organization criteria;^10^ high-risk or intermediate-2 MF risk, as defined by the International Prognostic Scoring System,^11, 12^ or intermediate-1 MF risk associated with symptomatic splenomegaly/hepatomegaly and/or unresponsive to available therapy; life expectancy ⩾12 weeks; Eastern Cooperative Oncology Group performance status ⩽2; and adequate organ function within 7 days prior to start the study treatment.

Patients were excluded if they had received chemotherapy, immunomodulatory drug therapy, immunosuppressive therapy, corticosteroids>10 mg/day prednisone or equivalent or erythropoietin within 2 weeks prior to the study treatment onset; incomplete recovery from surgery or radiotherapy within the 4 prior weeks; previous treatment with doxorubicin at cumulative doses⩾450 mg/m^2^; history of prior malignancies within last 3 years (except for early-stage basal cell or squamous cell skin cancer, cervical intraepithelial neoplasia, cervical carcinoma *in situ* or superficial bladder cancer); grade >2 myopathy or any clinical situation causing a persistent elevation of creatine phosphokinase; acute infection; and active liver disease, or any other unstable or serious medical condition (for example, uncontrolled arterial hypertension, myocardial infarction, cerebrovascular accident, valvular heart disease, symptomatic arrhythmia, so on). Patients were also excluded if they were pregnant or breast-feeding women, or if they were not using appropriate contraceptive measures.

### Treatment

Plitidepsin was given at the dose of 5 mg/m^2^ intravenously (i.v.) over 3 h on days 1 and 15 every 4 weeks (q4wk), for a maximum of 6 cycles. Prophylactic medication 20–30 min before each plitidepsin infusion consisted of dexamethasone 8 mg i.v., ondansentron 8 mg i.v. (granisetron 3 mg i.v. preferred when available), diphenhydramine hydrochloride 25 mg i.v., and ranitidine 50 mg i.v., or their equivalents. Additional prophylactic medication (metoclopramide and/or extended oral ondansetron) could be used if required. Plitidepsin treatment could be continued for more than six cycles when considered of clinical benefit for the patient.

A maximum of two plitidepsin dose reductions (from 5.0 to 4.0, then to 3.2 mg/m^2^) were allowed in case of any of the following events: grade 4 neutropenia lasting>7 days or accompanied by infection/fever; grade 4 thrombocytopenia lasting>7 days or accompanied by major bleeding; grade⩾3 nausea/vomiting or diarrhoea refractory to standard therapy; grade⩾3 muscular toxicity; grade⩾2 peripheral neuropathy; grade⩾3 transaminase increase>2 weeks, or any toxicity causing a dose delay of 1–2 weeks; grade⩾2 direct bilirubin increase; grade⩾3 CPK increase; or any other grade⩾3/4 non-haematological toxicity related to the study treatment (excluding grade⩾3 hypersensitivity reactions, grade 3 asthenia/ fatigue<5 days or grade 3 diarrhoea <1day).

### Efficacy assessment

The primary efficacy endpoint was response rate (RR) according to the International Working Group for Myelofibrosis Research and Treatment consensus criteria.^13^ Thus, a confirmed response included complete remission or partial remission, or clinical improvement that persisted for a minimum 8-week period. Efficacy was evaluated at the beginning of each plitidepsin cycle, independently of dose delays, up to 6 cycles of treatment. Progression-free survival and overall survival were also assessed as exploratory efficacy parameters.

### Safety assessment

Safety was evaluated in all patients who received at least one plitidepsin infusion, complete or incomplete, by assessment of adverse events (AEs), clinical laboratory test results, physical examinations and vital signs. AEs were recorded and coded with the Medical Dictionary for Regulatory Activities, v.12.0. AEs and laboratory values were graded according to the National Cancer Institute-Common Toxicity Criteria for Adverse Events NCI-CTCAE, v. 4.0. All patients were followed until recovery from any plitidepsin-related AE.

### Statistical methods

A Simon's optimal two-stage design^14^ was adopted. In a first stage, a minimum of 10 evaluable patients were to be accrued to test the null hypothesis, Ho: RR⩽15% versus Ha: RR⩾35% (alpha⩽0.1 and beta⩽0.1). At this first step, the largest RR to consider the study treatment as ineffective was 10%, and the smallest RR to consider the treatment worthy of further study was 20%. If the latter occurred, 35 additional evaluable patients were to be recruited. An RR of at least 22.2% in the total of 45 patients was required to conclude that the study treatment was effective.

Descriptive statistics were used for this study. Non-continuous variables are described in frequency tables using counts and percentages. Continuous variables are described by median, minimum and maximum. Binomial exact estimator and its 95% CI was calculated for the evaluation of the main endpoint (RR according to International Working Group for Myelofibrosis Research and Treatment) and other categorical efficacy variables (for example, progression-free survival and progression-free survival at fixed time points).

## Results

### Preclinical studies: effects of plitidepsin in cellular models

**Figure 1:**
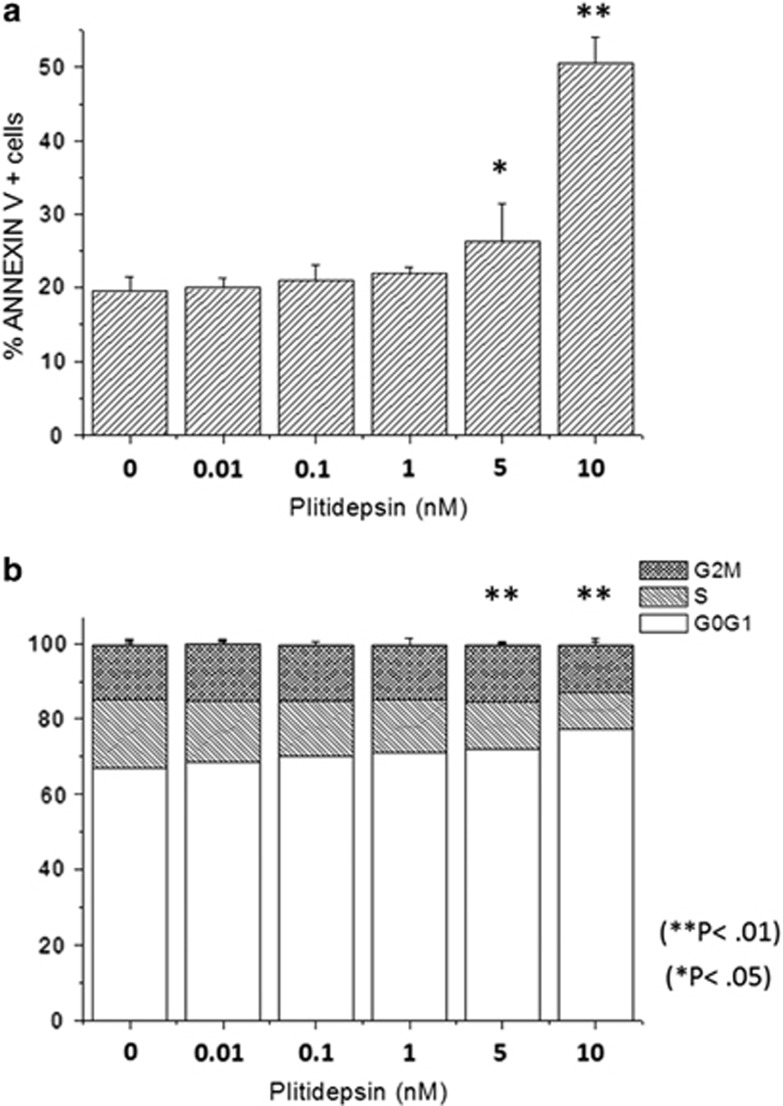
Effect of plitidepsin on cell death and cell cycle in SET2 cells. In (**a**), the percentage of Annexin V-positive cells was measured with Annexin V/propidium iodide staining and flow cytometry in cultures of SET2 cells that had been exposed to varying amount of plitidepsin for 48 h; cells incubated without the drug served as control. *P<0.05; **P<0.01. In (**b**), the frequency of cells in the G0/G1, S and M phase of the cell cycle was measured by flow cytometry after propidium iodide staining of SET2 cells that had been exposed to plitidepsin for 18 h, compared with control cells with vehicle. Results shown are the mean±s.d. of three independent experiments.

We first measured the growth inhibitory activity of plitidepsin in both a short-term liquid proliferation and a clonogenic assay. The IC_50_ values are reported in Table 1. We observed that the proliferation rate of HEL, UKE-1 and SET2 cells was inhibited at very-low nanomolar concentrations of plitidepsin, yet comparable with K562 cells; on the other hand, colony formation by UKE-1 and SET2 cells was inhibited at 3–5-fold lower plitidepsin concentrations compared with K562 cells. Murine Ba/F3 *JAK2*V617F cells resulted significantly more sensitive to plitidepsin than the wild-type counterpart in liquid assays (Table 1). Overall, these data indicate that plitidepsin inhibits proliferative activity of JAK2V617F-mutated cells at very-low nanomolar concentrations. The SET2 cell line only was later employed for assessing the effects of plitidepsin on cell cycle and apoptosis. The proportion of SET2 cells undergoing cell death was determined by Annexin V staining. As shown in Figure 1a, treatment with plitidepsin resulted in a dose-dependent, statistically significant increase of Annexin V-positive cells from 19.0±2.1–25.0±3.7% (*P*<0.05) and 49.0±2.0% (*P*<0.01) at 1 and 5 nm, respectively. We found that plitidepsin caused a dose-dependent accumulation of SET2 cells in the G0/G1 phase of the cell cycle from 65.5±3.5–71.5±3.3% at 5 nm (*P*<0.05) and 78.0±5.3% at 10 nm (*P*<0.01) (Figure 1b). Similar results were obtained with HEL cells (not shown).

The effects of plitidepsin on the clonogenic potential of haematopoietic progenitors from patients with myeloproliferative neoplasms were assessed by using a semisolid medium. For this purpose, CD34+ cells from JAK2V617F mutated (*n*=3) or *JAK2* wild-type (*n*=2) PMF patients, or healthy controls (*n*=5), were cultured in the presence of cytokines supporting the growth of BFU-E, CFU-G/GM or CFU-Mk. The drug was added once at the beginning of culture at increasing concentrations up to 5 nm, and the IC_50_ was calculated in comparison with the vehicle only. We found that the formation of all colony types from PMF cells was inhibited at a significantly lower concentration of plitidepsin compared to healthy controls; the IC_50_ values for BFU-E, CFU-GM and CFU-Mk were 8.7±2.3, 8.2±3.5 and 1.7±0.9 nm, respectively, in healthy controls versus 1.1±0.6 nm, 1.6±0.4 and 0.4±0.1 nm in PMF subjects; all the differences were statistically significant (*P*<0.01).

**Figure 2:**
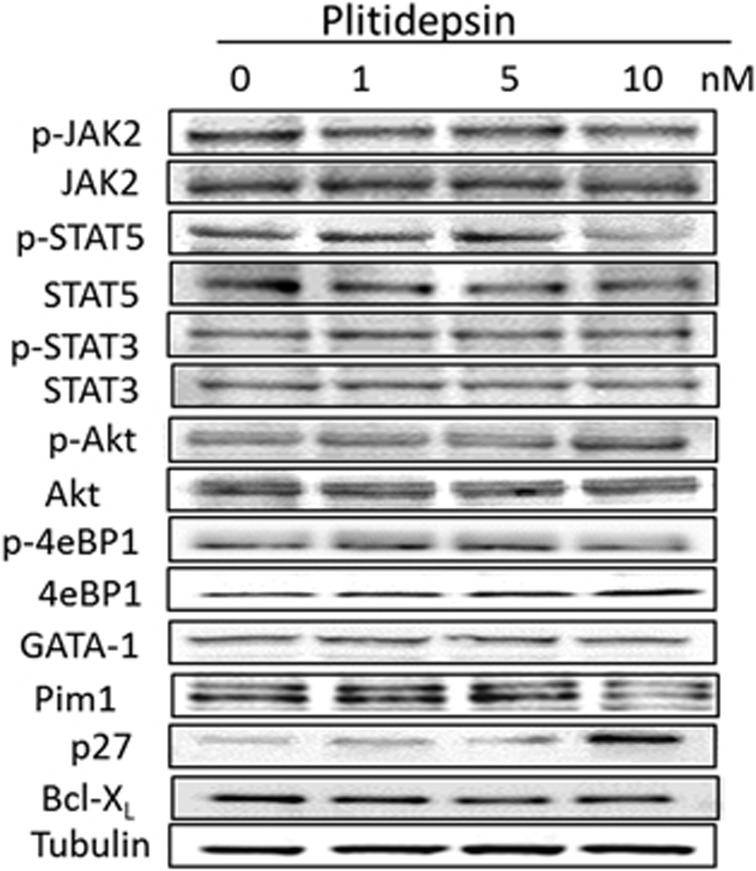
Effect of plitidepsin on the total protein expression and protein phosphorylation of selected downstream signalling proteins in SET2 cells. SET2 cells were incubated for 24 h with varying amount of plitidepsin, as indicated. Total and phosphorylated proteins were assayed by using specific antibodies and revealed by western blotting. Shown is one representative of at least three independent experiments for each protein target.

**Figure 3:**
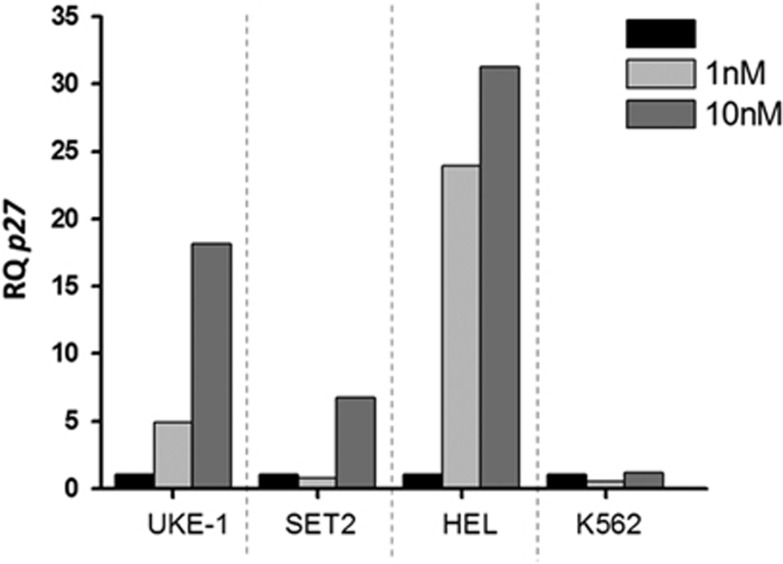
Effect of plitidepsin on P27 expression in human hematopoietic cell lines. Human cell lines were incubated with plitidepsin (1–10 nm) or in its absence (black columns) for 24 h. P27 mRNA expression was evaluated by real-time PCR and expressed as the relative quantity versus control cells.

To evaluate the effects on plitidepsin on downstream targets, we used western blot analysis in extracts of SET2 cells that had been exposed to varying concentration of the drug for 24 h. We failed to observe any significant modulation in the levels of total and phosphorylated forms of proteins involved in JAK/STAT signalling such as JAK2, STAT5, STAT3, as well as Akt and 4eBP1, GATA-1, Pim1 and Bcl-xL (Figure 2). On the other hand, we found a significant upregulation of p27 at the highest dose (10 nm); such an increase was due to plitidepsin acting at the transcriptional level since the amount of p27 mRNA measured by real-time quantitative PCR increased significantly in all myeloproliferative neoplasm-derived cells exposed to the drug (Figure 3). Of note, K562 appeared unresponsive to plitidepsin at this regard.

**Figure 4:**
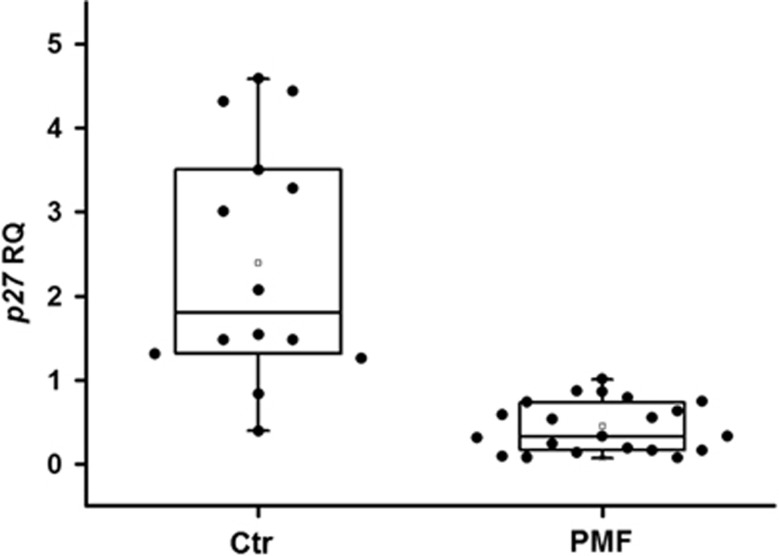
P27 mRNA levels in CD34+ cells. CD34+ from PMF patients and healthy controls were isolated from peripheral blood and analysed for p27 expression with real-time PCR. Results were expressed as relative quantity by using an RNAse P for normalisation. The difference between the two groups was highly significant (*P*<0.001).

Since low p27 cellular levels have been associated with response to plitidepsin in several cancer cells, we measured the levels of p27 mRNA in CD34+ cells from PMF patients compared with controls. As shown in Figure 4, we found that the p27 mRNA content was significantly reduced in patients' cells as compared with healthy controls; however, exposure to up to 10 nm plitidepsin of CD34+ cells from three PMF patients resulted in minimal changes in p27 mRNA levels (not shown).

### Phase II clinical trial

#### Patient characteristics

A total of 12 patients were included and treated with plitidepsin between 8 July 2010 and 6 April 2011. Their demographic and baseline characteristics are summarised in Table 2. At time of diagnosis, 5 patients (42%) had PMF, 3 (25%) had post-PV MF and 4 (33%) had post-ET MF. At the time of study entry, most patients (*n*=9, 75%) had high-risk disease according to International Prognostic Scoring System. The spleen was palpable in all patients, with splenomegaly⩾10 cm in 8 patients (67%). Hepatomegaly was present in 4 patients. All 12 patients had anaemia, mostly grade 2/3 (83%). Leucocytosis was present in 5 patients (42%), thrombocytosis in one patient, and both abnormalities in another patient. One patient had received one platelet transfusion, and 10 patients (83%) had received a median of 2 (range, 1–4) units of packed red blood cells (RBCs) within the 28 days prior to study entry. Bone marrow biopsies were performed in 11 patients and showed increased cellularity in 7 patients (64%), while 4 patients (36%) had decreased cellularity. Eleven patients (92%) had received prior immunomodulating and/or antineoplastic agents, most often hydroxycarbamide (50%) and thalidomide (42%). Four patients (33%) had received anti-anaemic preparations and one patient had undergone splenic radiation therapy.

#### Treatment and dosing

A total of 30 plitidepsin cycles were administered with a median number of two cycles per patient (range, 1–4). Median cumulative dose was 20.1 mg/m^2^ (range, 5.3–39.9 mg/m^2^), median dose intensity was 2.2 mg/m^2^/week (range, 1.3–2.5 mg/m^2^ per week), and median relative dose intensity was 86.8% (range, 52.6–100.7%).

A total of four cycles were delayed in four patients (that is, 40% of the 10 patients who received more than one cycle), with a median duration of 13.5 days (range, 7–15 days). Dose omissions occurred in 2 cycles. All these dose delays/omissions were due to causes unrelated to the study treatment: left ankle fracture, grade 4 neutropenia because of the disease, grade 3 oesophageal varices haemorrhage, grade 2 blood creatinine increase and grade 2 bronchitis in the case of dose delays, and grade 2 rash macular and grade 3 gastrointestinal bleeding in the case of dose omissions. No dose reductions were required.

#### Efficacy

One of the 12 treated patients was excluded from analysis of the primary efficacy endpoint. This patient received one complete infusion of plitidepsin in Cycle 1, and had the second infusion interrupted because of plitidepsin-related grade 3 chest and epigastric pain. Although the episode resolved a day later, the patient refused to continue treatment and had no disease evaluations done.

The primary analysis of best response according to International Working Group for Myelofibrosis Research and Treatment in the 11 evaluable patients showed clinical improvement in one patient (9.1%), stable disease in 9 patients (81.8%), and progressive disease in one patient (9.1%). Characteristics of patients with clinical improvement or stable disease are shown in Table 3. The patient with clinical improvement was red cell transfusion-dependent at baseline and converted to transfusion-independent with treatment that persisted for more than 8 weeks. No partial or complete remissions were observed. Therefore, RR according to International Working Group for Myelofibrosis Research and Treatment was 9.1% (95% CI, 0.2–41.3%). Median progression-free survival in the 11 evaluable patients was 4.6 months (95% CI, 1.4–4.6 months). Median overall survival had not been reached at cut-off date.

Eight patients underwent a short-lasting improvement of splenomegaly, with maximum size reductions occurring during the first two cycles of treatment (Table 3).

#### Safety

The safety population included all 12 treated patients. Table 4 shows the main worst grade plitidepsin-related AEs; the most common were fatigue, nausea, vomiting and muscular weakness. Three patients had grade 3 AEs in one cycle each, which comprised fatigue, upper abdominal pain and chest pain. No grade 4 drug-related AEs occurred.

Three patients had isolated grade 1/2 prolonged electrocardiogram (ECG) QT interval of unknown relationship to plitidepsin in a total of 7 cycles. One of the patients, diagnosed with high-risk post-ET MF, had displayed abnormal ECG and chest exam (2/6 ejection murmur) at study entry. A second patient, diagnosed with intermediate-2 PMF, had not reported previous cardiac complications or risk factors. The third patient, diagnosed with intermediate-1 post-PV MF, had asymptomatic degenerative aortic valvulopathy and mitral insufficiency at baseline and history of transient ischaemic attacks.

The most common haematological abnormality irrespective of relationship with plitidepsin treatment was anaemia, which occurred in all patients at all cycles, followed by lymphopenia and thrombocytopenia (Table 4).

All biochemical abnormalities were grade 1/2, and the only with effect on treatment was one case of grade 2 creatinine increase, which caused dose delay in one cycle (Table 4).

Two patients discontinued plitidepsin administration due to events unrelated to the study treatment: grade 4 thrombocytopenia, and grade 3 pulmonary oedema, bronchopneumonia and acute myocardial infarction.

## Discussion

### Preclinical evaluation

Although the mechanism of action of plitidepsin remains to be fully characterised, several targets have been identified in various cellular models.^15^ Plitidepsin caused a dose-related arrest of cell cycle and cell apoptosis following the induction of an early oxidative stress, the activation of Rac1 GTPase and the inhibition of protein phosphatases. The block of cell cycle at G_0_/G_1_ is largely dependent on the activity of the CdK inhibitor p27, and an inverse correlation between the expression level of p27 and the response to plitidepsin has been demonstrated in human sarcoma cell lines.^16^ Inhibition of cell viability occurs through the mitochondrial apoptotic pathway, release of cytochrome c, PARP cleavage and chromatin fragmentation.^17, 18^ A sustained activation of members of the MAPK family, including the serine/threonine kinases JNK and p38 and possibly ERK, is rapidly induced by plitidepsin in several tumour cell models and at least in part it is mediated by Rac1,^19, 20^ a member of the guanine triphosphatase family downstream of the canonical Wnt signaling.^21^ Finally, plitidepsin has anti-angiogenic properties and inhibits spontaneous and vascular endothelial growth factor- and FGF-2-induced angiogenesis in the chick allantoid assay.^22, 23, 24^ In a previous work using the GATA-1low mouse model of MF,^7^ we showed that the MF trait of the mice could be efficiently corrected by plitidepsin that, by restoring the expression of Gata1 and p27(Kip1) in Gata1-low haematopoietic cells, corrected the proliferation of marrow progenitor cells *in vitro* and maturation of megakaryocytes *in vivo* through a reduction of the levels of transforming growth factor-beta and vascular endothelial growth factor abnormally released by immature Gata1low megakaryocytes in the bone marrow microenvironment. Microvessel density, fibrosis, bone growth and marrow cellularity were normalised after plitidepsin treatment of the mice and extramedullary haematopoiesis did not develop in liver; notwithstanding, the abnormally reduced CXCR4 expression in Gata-1(low) progenitor cells was not improved by plitidepsin. These preclinical results suggested that plitidepsin had the potentiality to improve the MF phenotype of GATA-1low mice, justifying further clinical development.^25^ In the current study, we produce evidence that plitidepsin at low nanomolar concentrations exerted potent antiproliferative activity and induced cell cycle arrest and apoptosis in different cellular models of JAK2V617F mutation and also prevented colony formation by primary myeloproliferative neoplasm CD34+ cells. In the cell line models, the effects of plitidepsin were consistent with an upregulation of p27; however, although the level of p27 mRNA were definitely lower in MF CD34+ cells than in control cells, plitidepsin failed to normalise those levels in the human samples. Overall, these data confirm the potent cytotoxic activity of plitidepsin even against cells of myeloproliferative neoplasms, although evidence of a preferential activity of the drug compared to control cells was modest at all.

### Clinical evaluation

The exploratory phase II trial that we report in this manuscript was designed to evaluate the efficacy and safety of plitidepsin in patients with PMF, post-PV MF or post-ET MF. Plitidepsin has shown antitumour activity in several solid tumours^26, 27^ as well as in some malignant haematological disorders.^28, 29^ The schedule (q4wk) and dose (5 mg/m^2^ 3-h i.v. infusion) used in this phase II study had been effective and with an adequate benefit/risk ratio in previous studies conducted in patients with various solid tumours or multiple myeloma^26, 27, 28, 30^

In the first stage of this trial, RR was 9.1%, which was lower than the minimum protocol-defined threshold (20%) required for further assessment of this regimen in this disease. Therefore, we concluded that the current treatment regimen had low activity in this population of patients with PMF, post-PV MF or post-ET MF. Drugs such as hydroxyurea and interferon-alpha have modest activity in controlling splenomegaly and leucocytosis in patients with PMF, and favourable responses to thalidomide and lenalidomide, chiefly in the form of improved haemoglobin and platelet counts, have been reported in a small subset of patients.^31, 32^ Ruxolitinib (a JAK-1/2 inhibitor) was recently approved for the treatment of intermediate and high-risk MF, including PMF, post-PV MF or post-ET MF, with ⩾35 percent reduction in splenic volume in 41.9% of patients, which was maintained for 48 weeks in the majority of patients with such a response.^5, 33^ In the current phase II exploratory trial, only one patient had confirmed disease response (anaemia improvement), whereas most patients had stable disease as best response. Nevertheless, in the indication evaluated, stable disease frequently equates with highly symptomatic disease for most patients, usually characterised by poor quality of life. Finally, after taking into account toxicity, quality/duration of response, patterns of treatment failure and the activity observed with other drugs, it was decided to terminate the study before going into the second stage.

Plitidepsin was generally well tolerated and showed manageable toxicity when administered to this population of patients with PMF, post-PV MF or post-ET MF. The most common toxicities found in this study (fatigue, nausea/vomiting, and muscular weakness) were consistent with those previously reported with plitidepsin.^26, 27, 28, 30, 34, 35, 36^ In general, these toxicities were not dose-limiting in the majority of patients, and were manageable by appropriate dose modifications or administration delays. Three cases of grade 1/2 prolonged QT ECG of unknown relationship with plitidepsin were reported. Two of these three patients had cardiac risk factors at baseline.

In conclusion, due to the modest antitumour activity reported, plitidepsin given at a dose of 5 mg/m^2^ i.v. over 3 h on Day 1 and 15 q4wk to patients with PMF, post-PV MF or post-ET MF was considered as not worthy of further clinical evaluation. The toxicity profile of plitidepsin at this schedule of administration was consistent with that observed with plitidepsin in other clinical trials in patients with solid tumours and haematological disorders. Preclinical results showed potent cytotoxic activity of plitidepsin against myeloproliferative neoplasms that were not confirmed in this exploratory phase II trial. A plausible reason could be that the efficacy observed in mice was seen in a monogenic disorder (GATA-low) that mirrors just one late mechanism of the disease (megakaryocytes proliferation associated with deposition of fibrosis)^37^ but does not reflect the likely multigenic, complex pathogenesis of a stem cell disorder (not only megakaryocytes) as is human MF.

## Figures and Tables

**Table 1:** Determination of plitidepsin IC50 in human and murine JAK2V617F-mutated cell lines and controls

*Cell line*	*IC*_*50*_ *(nm)*	
	*WST-1 assay*	*Clonogenic assay*
K562	1.50±0.10	2.70±0.30
HEL	1.00±0.30	1.50±0.05
SET2	1.00±0.30	0.80±0.02**
UKE-1	2.40±0.20	0.50±0.03**
Ba/F3 JAK2 wild-type	0.40±0.03	ND
Ba/F3 JAK2 V167F	0.03±0.01**	ND

Abbreviations: IC_50_, plitidepsin concentration that reduced colony number to 50% that measured in control dishes with vehicle only; ND, not done.

IC_50_ value was calculated using both short-term proliferation assay in liquid cultures and long-term clonogenic assay in agar. Control murine Ba/F3 wild-type cells were maintained in the presence of IL-3. **P<0.01.

**Table 2:** Demographic and baseline characteristics (*n*=12)

*Characteristic*	n	*%*
*Gender*		
Male	5	42
Female	7	58
Age (years)	69.5 (59–78)	
*ECOG PS*		
0	4	34
1	7	58
2	1	8
*Myelofibrosis*		
Primary myelofibrosis	5	42
Post-polycythaemia vera	3	25
Post-essential thrombocythaemia	4	33
*IPSS risk*		
High	9	75
Intermediate-2	2	17
Intermediate-1	1	8
*Laboratory studies/physical examination at enrolment*		
Spleen size by ultrasound (cm^2^)^a^		
<100 cm^2^	1	12
100–200 cm^2^	3	38
>200 cm^2^	4	50
Bone marrow biopsy cellularity^b^		
Increased	7	64
Decreased	4	36
Haemoglobin (g/dl)	9.1 (8.0–11.9)	
Platelet count ( × 10^9^/l)	140.5 (32.0–677.0)	
Leukocyte count ( × 10^9^/l)	10.4 (1.8–51.2)	
LDH (x ULN)	2.5 (0.8–12.0)	
*Previous therapy*		
Corticosteroids		
Prednisone	7	58
Antineoplastic/immunomodulating		
Anagrelide	3	25
Etanercept	1	8
Hydroxicarbamide	6	50
Lenalidomide	2	17
Rituximab	1	8
Thalidomide	5	42
Other immunosupressants	5	42
Anti-anaemic		
Darbepoetin alpha	2	17
Epoetin alpha	2	17
Radiotherapy (spleen)	1	8

Abbreviations: ECOG PS, Eastern Cooperative Oncology Group performance status; IPSS, International Prognostic Scoring System; LDH, lactate dehydrogenase; ULN, upper limit of normal.

Data shown are *n* of patients (%) except for age and laboratory data (median and range).

a Spleen size by ultrasound was missing in four patients. Palpable spleen size was as follows: <10 cm (*n*=4), 10–19 (*n*=7) and ⩾20 cm (*n*=1).

b Assessment not done in one patient.

**Table 3:** Treatment response characteristics of patients treated with plitidepsin

*Gender/age (years)*	*MF type*	*ECOG PS BL/WPC*	*Plitidepsin cycles*	*Best response*^a^	*PFS /OS (months)*	*Plt/RBC transfusion (units)*		*Spleen reduction*^b^ *(%)*
						*Baseline*	*On treatment*	
Male/77	Post PV	1/2	4	Clinical improvement^c^	4.6/4.6	0/2	0/0	21.4
Female/67	Post ET	1/2	1	SD	0.9+/1.7+	0/1	1/1	0.0
Female/68	Post ET	1/2	4	SD	3.6+/4.5+	0/2	0/2	22.2
Female/64	PMF	1/1	2	SD	1.0+/1.7+	0/2	0/3	11.1
Female/67	PMF	0/1	3	SD	1.8+/5.1+	0/1	0/2	ND
Male/72	PMF	1/3	2	SD	2.3+/2.3+	0/2	0/4	35.0
Male/73	Post PV	1/1	2	SD	1.9+/2.1+	1/2	0/10	53.3
Male/71	PMF	2/2	2	SD	2.0+/2.0+	0/2	0/7	10.5
Male/64	PMF	0/0	3	SD	2.8+/3.8+	0/0	0/10	7.7
Female/78	Post PV	0/1	2	SD	1.8+/4.8+	0/0	0/0	22.2

Abbreviations: ECOG, Eastern Cooperative Oncology Group; IWG-MRT, International Working Group for Myelofibrosis Research and Treatment; MF, myelofibrosis; ND, not determined; OS, overall survival; PFS, progression-free survival; Plt, platelet; post-ET, post-essential thrombocythaemia; post-PV, post-polycythaemia vera; PMF, primary myelofibrosis; PS, performance status; RBC, red blood cell; SD, stable disease; WPC, worst per cycle.

a Best response as per IWG-MRT.

b Maximal reduction from baseline by spleen palpation, which was reached within the first two cycles and persisted less than 8 weeks in all patients measured.

c Time to response was 1.9 months.

+: Censored data.

**Table 4:** Main worst grade plitidepsin-related adverse events (⩾10% of patients or cycles)

*Adverse event*	*Per patient (*n*=12)*				*Per cycle (*n*=30)*			
	*Grade 1/2*		*Grade 3/4*		*Grade 1/2*		*Grade 3/4*	
	n	*%*	n	*%*	n	*%*	n	*%*
*Haematological*^a^								
Anaemia	3	25	9	75	13	43	17	57
Leukopenia	1	8	4	33	2	7	9	30
Lymphocytosis	3	25	—	—	5	17	—	—
Lymphopenia	5	42	4	33	13	43	6	20
Neutropenia	2	17	3	25	3	10	7	23
Thrombocytopenia	4	33	4	33	10	33	6	20
*Non-haematological*^a^								
ALT increase	8	67	—	—	10	35	—	—
AP increase	8	67	—	—	21	72	—	—
AST increase	8	67	—	—	14	48	—	—
CPK increase	4	33	—	—	4	14	—	—
Creatinine increase	6	50	—	—	11	38	—	—
Diarrhoea	2	17	—	—	4	13	—	—
ECG QT interval prolonged	3	25	—	—	7	23	—	−
Fatigue	4	33	2	17	6	20	2	7
Muscular weakness	3	25	—	—	4	13	—	—
Nausea	4	33	—	—	5	17	—	—
Vomiting	3	25	—	—	3	10	—	—

Abbreviations: ALT, alanine aminotransferase; AP, alkaline phosphatase; AST, aspartate aminotransferase; CPK, creatine phosphokinase; ECG, electrocardiogram.

Apart from the adverse events shown in this table, one patient each had grade 3 abdominal pain upper and grade 3 chest pain in one cycle each.

a Laboratory abnormalities are shown irrespective of relationship with the plitidepsin treatment.

## Notes

AP, AT, PG, CB, NB and AMV declare no competing financial interests. JR, SE, IP and VA are employees of PharmaMar.
