# Supplementary material for: Evaluation of plitidepsin in patients with primary myelofibrosis and post polycythemia vera/essential thrombocythemia myelofibrosis: results of preclinical studies and a phase II clinical trial
Source: Blood Cancer J. 2015 Mar 13;5(3):e286–. doi: 10.1038/bcj.2015.5 (PMC4382667; doi:10.1038/bcj.2015.5)
Supplement: Supplementary Information [file bcj20155x1.doc]

**SUPPLEMENTAL MATERIAL**

**MATERIALS AND METHODS**

**Reagents**

Plitidepsin was provided by PharmaMar; it was dissolved in DMSO and stored in aliquots at -20°C. Antibodies against phospho(p)-STAT5 (Tyr694), total STAT5, pAKT (Ser473), total AKT, p-4EBP1 (Thr70), total 4EBP1, p-mTOR (Ser2448), total mTOR,  p-JAK2 (Tyr1007/1008), total JAK2, p-STAT3 (Tyr705), total STAT3, p27, Pim1, Bcl-X_L_ were from Cell Signaling Technology (Danvers, MA, US). Anti-human tubulin antibody was from Santa Cruz Biotechnology (Santa Cruz, CA, US). Recombinant human IL-3, IL-6, SCF, TPO and EPO were purchased from Miltenyi Biotec (Gladbach, Germany).

**Cell lines** **and cell culture**

The HEL, SET2 and K562 human cell lines were purchased from the German Collection of Microorganisms and Cell Cultures (DSMZ, Braunschweig, Germany). UKE1 cells were kindly provided by Dr. W. Fiedler (Hamburg, Germany), while murine BaF/3 cells overexpressing *JAK2* wild-type (wt) or *JAK2*V617F were donated by R. Skoda (Basel, Switzerland). Human cell lines were cultured in Iscove modified Dulbecco (IMDM) (UKE1) or RPMI 1640 medium (HEL, SET2 and K562). Ba/F3 cells were cultured in RPMI 1640 medium with or without 1ng IL3/mL. All media were supplemented with 10% fetal bovine serum (FBS; Lonza, Belgium) (20% for SET2 cells), antibiotics and L-glutamine (200 mM in 0.85% NaCL solution). Medium for UKE-1 also contained 10% horse serum (StemCell Techn. Inc, Canada) and 50 µM hydrocortisone (Sigma-Aldrich).

To measure the drug-induced inhibition of cell growth, we used both a short-term microwell proliferation assay and a long-term clonogenic assay in agar. Cellular proliferation was evaluated using the colorimetric WST-1 Assay (Roche, USA) in a 96 well microtiter plate reader (BioTek, USA). All cell lines were plated at 2 ·10⁴ cells/well in culture medium, in a 100 µl-volume in 96-well plates. Cells were treated with compound, or vehicle, 24 h after plating and grown for additional 48 h; then WST-1 reagent (10 µl/well) was added, cells were incubated at 37 °C in 5% CO2 for 2 to 4 hr, and absorbance was measured at 450 nm using a 620 nm reference wavelength filter. For clonogenic assay, 5x10^3^ /mL cells were plated in 0.5% agar in IMDM or RPMI-1640 medium, supplemented with FBS, and variable amount of the drug (or an equivalent volume of vehicle in control plates) were added once at the beginning of culture. Plates were incubated at 37°C in a fully-humidified atmosphere with 5% CO_2_ for 7 days, and colonies were enumerated by inverted microscopy. Data were normalised to percent of the control, and IC_50_ value (i.e., the concentration of drug that reduced cell proliferation or colony number to 50% that measured in control dishes with vehicle) was calculated.

**Primary cells**

Samples of peripheral blood (PB) or bone marrow (BM) were collected from patients with PMF, diagnosed according to the 2008 World Health Organization (WHO) criteria, under a protocol approved by Institutional Review Board of Azienda Ospedaliera-Universitaria Careggi and after obtaining an informed consent. Healthy donors of hematopoietic stem cells for transplantation purposes agreed to donate excess CD34^+^ cells, after providing informed consent. Research was carried out according to the principles of the Declaration of Helsinki. PB or BM mononuclear cells were separated over a Ficoll-Hypaque gradient (Lymphoprep, Nycomed Pharma; Oslo, Norway). CD34^+^ cells were immunomagnetically selected and characterised as described [^1^](#_ENREF_1). The *JAK2*V617F mutational status was determined by a quantitative real-time PCR assay in purified granulocytes, as described [^2^](#_ENREF_2).

**Assessment of apoptosis and cell cycle**

Cells were incubated with different amounts of the drug and analysed 48 hours later for determination of apoptosis and 18 hr later for cell cycle analysis. Quantification of apoptotic cells was accomplished by flow cytometry with a FACScan instrument (Becton-Dickinson, San Jose, CA, USA) using the Annexin-V-FLUOS Staining kit (Roche, USA). Usually 5x 10^5^ cells were used for labeling with Annexin-V-FLUOS and propidium iodide (PI) in binding buffer, according to manufacturer’s instructions; at least 20,000 events were obtained. For cell cycle distribution, 1x10^6^ cells were treated with ethanol 95%, RNase 10 μg/mL, and propidium iodide 50 mg/mL, and then analyzed by flow cytometry. Data analysis was performed using ORIGIN software (V7.5, OriginLab Northampton, MA, [http://www.originlab.com](http://www.originlab.com/)).

**Colony assays**

Mononuclear cells from MPN patients or control subjects were plated at 1x10^5^/mL for the growth of BFU-E and CFU-GM in methylcellulose (MethoCult; StemCell Technologies, Vancouver, Canada) added with cytokines: SCF 50ng/mL, IL-3 10ng/mL, IL-6 10ng/mL, GM-CSF 10ng/mL, G-CSF 10ng/mL and EPO 3U/mL. For the growth of CFU-Mk, CD34^+^ cells were plated at 3x10^3^/mL in a 24-well plate in Megacult Collagen and medium with lipids (StemCell Technol.) in presence of cytokines: Thrombopoietin 50ng/mL, IL-3 10ng/mL, IL-6 10ng/mL. Colonies were enumerated on day 12-14 according to standard criteria.

**Cell lysis and SDS-PAGE Western blotting**

Cells were resuspended in RIPA lysis buffer (50mM pH 7.4 Tris-HCl, 150mM NaCl, 1% NP-40, 1mMEDTA) containing a proteinase inhibitor cocktail (Halt Protease Inhibitor Cocktail Kit, PIERCE, Rockford, IL, US), centrifuged to remove nuclei, and then subjected to sodium dodecyl sulphate polyacrylamide gel electrophoresis separation and western blotting onto Immunoblot PVDF membrane (BioRad, Hercules, CA, US), according to standard protocols. Membranes were probed with primary antibodies followed by horseradish peroxidase-conjugated anti-Ig antibody produced in rabbits (Sigma-Aldrich), and immunoreactive proteins were revealed by the ECL procedure (GE Healthcare, Little Chalfont, UK) on Hyperfilm ECL or with the Image Quant 350 apparatus (GE Healthcare).

**RNA isolation and Real-Time quantitative PCR (RTQ-PCR)**

Total RNA was purifed using Trizol (Invitrogen-Life Technologies, Paisley, UK), and the RNA concentration and purity/integrity was determined with NanoDrop ND-1000 spectrophotometer (NanoDrop Techn., Wilmington, DE, USA). One microgram of RNA was reverse transcribed using High Capacity cDNA Archive Kit (Applied Biosystems, Foster City, CA). RT-QPCR reactions were carried out with the TaqMan Universal PCR Master Mix using ABI PRISM 7300 HT Sequence Detection System, and a TaqMan® Gene Expression Assay (P27 *CDKN1B* Applied Biosystems), in triplicate. Gene expression profiling was achieved using the Comparative cycle threshold (C_T_) method of relative quantitation using VIC-labeled *RNaseP* probe as the housekeeping gene (ΔC_T_).

**Statistical methods**

Comparison between groups was performed by the Mann-Whitney *U* or Fisher test as appropriate, using the SPSS software (StatSoft, Inc., Tulsa, OK, [http://www.statsoft.com](http://www.statsoft.com/)) or ORIGIN software (v. 7.5, OriginLab Northampton, MA, [http://www.originlab.com](http://www.originlab.com/)) for computation. The chosen level of significance from two-sided tests was P<0.05.

**References**

1. Vannucchi AM, Pancrazzi A, Guglielmelli P, Di Lollo S, Bogani C, Baroni G*, et al.* Abnormalities of GATA-1 in megakaryocytes from patients with idiopathic myelofibrosis. *Am J Pathol* **2005**; 167: 849-858.

2. Lippert E, Boissinot M, Kralovics R, Girodon F, Dobo I, Praloran V*, et al.* The JAK2-V617F mutation is frequently present at diagnosis in patients with essential thrombocythemia and polycythemia vera. *Blood* **2006**; 108: 1865-1867.
